# Supplementary material for: How Reliable Is Fluorescence-Guided Surgery in Low-Grade Gliomas? A Systematic Review Concerning Different Fluorophores
Source: Cancers (Basel). 2023 Aug 16;15(16):4130. doi: 10.3390/cancers15164130 (PMC10452554; doi:10.3390/cancers15164130)
Supplement: Supplementary file 1 [file cancers-15-04130-s001.zip › cancers-2504536-supplementary.pdf]

Search string

1) "low"[All Fields] AND ("grade"[All Fields] OR "graded"[All Fields] OR "grades"[All Fields] OR "grading"[All Fields] OR "gradings"[All Fields]) AND ("glioma"[MeSH Terms] OR "glioma"[All Fields] OR "gliomas"[All Fields] OR "glioma s"[All Fields]) AND ("aminolevulinic acid"[MeSH Terms] OR ("aminolevulinic"[All Fields] AND "acid"[All Fields]) OR "aminolevulinic acid"[All Fields] OR "5 ala"[All Fields] OR ("aminolevulinic acid"[MeSH Terms] OR ("aminolevulinic"[All Fields] AND "acid"[All Fields]) OR "aminolevulinic acid"[All Fields]) OR "gliolan"[All Fields] OR ("fluorescein"[MeSH Terms] OR "fluorescein"[All Fields] OR ("sodium"[All Fields] AND "fluorescein"[All Fields]) OR "sodium fluorescein"[All Fields]) OR ("indocyanine green"[MeSH Terms] OR ("indocyanine"[All Fields] AND "green"[All Fields]) OR "indocyanine green"[All Fields]) OR ("tozuleristide"[Supplementary Concept] OR "tozuleristide"[All Fields] OR "blz 100"[All Fields]) OR ("hypericin"[Supplementary Concept] OR "hypericin"[All Fields] OR "hypericins"[All Fields]))

2) "low"[All Fields] AND ("grade"[All Fields] OR "graded"[All Fields] OR "grades"[All Fields] OR "grading"[All Fields] OR "gradings"[All Fields]) AND ("glioma"[MeSH Terms] OR "glioma"[All Fields] OR "gliomas"[All Fields] OR "glioma s"[All Fields]) AND ("fluoresce"[All Fields] OR "fluoresced"[All Fields] OR "fluorescence"[MeSH Terms] OR "fluorescence"[All Fields] OR "fluorescences"[All Fields] OR "fluorescent"[All Fields] OR "fluorescently"[All Fields] OR "fluorescents"[All Fields] OR "fluoresces"[All Fields] OR "fluorescing"[All Fields] OR ("fluorophor"[All Fields] OR "fluorophore"[All Fields] OR "fluorophore s"[All Fields] OR "fluorophores"[All Fields] OR "fluorophoric"[All Fields] OR "fluorophors"[All Fields]))

3) "low"[All Fields] AND ("grade"[All Fields] OR "graded"[All Fields] OR "grades"[All Fields] OR "grading"[All Fields] OR "gradings"[All Fields]) AND ("glioma"[MeSH Terms] OR "glioma"[All Fields] OR "gliomas"[All Fields] OR "glioma s"[All Fields]) AND ((("intraop"[All Fields] OR "intraoperative"[All Fields] OR "intraoperatively"[All Fields]) AND ("fluoresce"[All Fields] OR "fluoresced"[All Fields] OR "fluorescence"[MeSH Terms] OR "fluorescence"[All Fields] OR "fluorescences"[All Fields] OR "fluorescent"[All Fields] OR "fluorescently"[All Fields] OR "fluorescents"[All Fields] OR "fluoresces"[All Fields] OR "fluorescing"[All Fields])) OR ("fluoresce"[All Fields] OR "fluoresced"[All Fields] OR "fluorescence"[MeSH Terms] OR "fluorescence"[All Fields] OR "fluorescences"[All Fields] OR "fluorescent"[All Fields] OR "fluorescently"[All Fields] OR "fluorescents"[All Fields] OR "fluoresces"[All Fields] OR "fluorescing"[All Fields]))
